# A Novel Treatment for Phthisis Proposed

**Published:** 1891-11

**Authors:** Thos. F. Turpin

**Affiliations:** Laredo, Texas


### A NOVEL TREATMENT FOR CONSUMPTION PROPOSED.

LAREDO, TEXAS, November 1, 1891.

*Editor Daniel's Texas Medical Journal, Austin, Texas;*

I want your opinion upon the following theoretical treatment of phthisis. Tell me first, has this ever been suggested by any one else (of this you are better able to judge than I), and then give me the benefit of your criticism:

My plan of treatment is this: Take a "pelone" dog, and after proper antiseptic precautions, join him, by a large skin flap, to the phthisical patient, and then envelop the dog in a plaster bandage, so as to prevent motion, and allow the flap (from the side or arm of the man) to grow to the corresponding part of the dog.

In a short time there will be an interchange of the blood currents of the two, and if, as is claimed, the blood of a dog is "immune" to phthisis, in a short time the "bacilli" will have been destroyed.

This will be a much more reasonable treatment than the very dangerous and temporary plan of injecting dog's blood into the patient's veins.

You remember, no doubt, the case reported some months ago of an attempt to graft a dog's bone upon a man. The operation was a failure, but it was stated that while the bone failed to unite, the skin flap united perfectly, and the temperature of man and dog were both affected.

Of course this idea will be laughed at, but if (a very large if, by the way) the blood of a dog, or any other animal, is really "immune" to the bacilli of consumption, the plan suggested would certainly be justifiable.

Write me what you think, and if I am the first to suggest this, publish all or part of this letter, and let us hear what the profession think of it. Very truly your friend,

THOS. F. TURPIN.

